# Novel sensory preconditioning procedures identify a specific role for the hippocampus in pattern completion

**DOI:** 10.1016/j.nlm.2016.02.006

**Published:** 2016-04

**Authors:** Tzu-Ching E. Lin, Natasha M. Dumigan, Mark Good, Robert C. Honey

**Affiliations:** School of Psychology, Cardiff University, Park Place, Cardiff CF10 3AT, UK

**Keywords:** Rat, Sensory associations, Pattern memory, Retrieval-mediated learning, Limbic system

## Abstract

Successful retrieval of a memory for an entire pattern of stimulation by the presentation of a fragment of that pattern is a critical facet of memory function. We examined processes of pattern completion using novel sensory preconditioning procedures in rats that had either received sham lesions (group Sham) or lesions of the hippocampus (group HPC). After exposure to two audio-visual patterns (AX and BY) rats received fear conditioning with X (but not Y). Subsequent tests assessed fear to stimulus compounds (e.g., AX versus BX; Experiment 1) or elements (A versus B; Experiment 2). There was more fear to AX than BX in group Sham but not group HPC, while there was more fear to A than B in group HPC, but not in group Sham. This double dissociation suggests that pattern completion can be based upon separable processes that differ in their reliance on the hippocampus.

## 1 Introduction

The hippocampus has been implicated in pattern completion, the process by which a stored representation for a pattern of stimulation can be retrieved by a subset of its elements (e.g., Leutgeb et al., 2007, Rolls, 2013). The nature of this process can be investigated using sensory preconditioning (SPC): where exposure to a pattern (AX; e.g., an audio-visual compound) can allow fear conditioned to X to be evident during A (e.g., Brogden, 1939, Rescorla, 1980). SPC is disrupted by lesions of hippocampus made prior to behavioral testing in both rabbits (Port, Beggs, & Patterson, 1987) and rats (Talk, Gandhi, & Matzel, 2002; see also, Eichenbaum, Mathews, & Cohen, 1989; but see, Honey & Good, 2000). However, the pattern completion processes that underpin SPC is often characterized in two ways: A could elicit fear *at test* through the operation of an (elemental) A-X-footshock associative chain (e.g., Hebb, 1943), the component links being forged during exposure to A with X (A-X) and conditioning with X (X-footshock). Alternatively, A and X could become linked to a separate configural memory (AX), with the later presentation of A eliciting fear to the extent that it activates the configural memory (i.e., AX) linked to shock *during conditioning* with X (e.g., Honey, Iordanova, & Good, 2014). Here, the role of the hippocampus in pattern completion was investigated using SPC procedures where these specific contributions of elemental and configural processes can be determined on an *a priori* basis.

Recent research identifies a role for configural processes in some SPC procedures. In one study, rats received exposure to AX and BY (two audio-visual compounds), and then X and Y were paired with footshock. During the test, there was more fear during the exposed compounds (AX and BY) than nonexposed compounds (AY and BX; Lin, Dumigan, Recio, & Honey, 2016). These results suggest that when X and Y were presented during conditioning they evoked the configural memories of AX and BY which became linked to shock (cf. Iordanova, Burnett, Good, & Honey, 2011; see also, Holland, 1981). Additional support for this analysis can be derived from the simpler observation that after exposure to AX and BY, conditioning with X results in greater fear to AX than BX (see Lin, Dumigan, Dwyer, Good, & Honey, 2013; see also, Ward-Robinson et al., 2005, Ward-Robinson and Hall, 1996). The associative chain analysis does not predict this outcome because A is only held to provoke more fear than B by dint of its capacity to activate X, and the presence of X with A and B will now mean that both compounds will have this capacity. Experiment 1 used this procedure (see Table 1) to examine whether lesions to the hippocampus disrupt SPC when the contribution of simple elemental processes has been counteracted and configural processes should be most apparent: AX is more similar to the configuration created during exposure and linked to shock during conditioning than is A alone. Experiment 2 used an identical procedure with the exception that A and B were presented alone during the test. Under these conditions, the A-X-shock chain can contribute to the level of fear to A, but the contribution of configural processes will be constrained by the similarity of A to the configural AX representation.

## 2 Materials and methods

### 2.1 Animals

Thirty-two male Lister hooded rats (*Rattus norvegicus*; supplied by Harlan Olac Ltd, UK) were used in two experiments: Experiment 1 (group Sham: *N* = 8; group HPC: *N* = 8; mean *ad libitum* weight: 421 g, range: 394–492 g), and Experiment 2 (group Sham: *N* = 8; group HPC: *N* = 8; mean *ad libitum* weight: 352 g, range: 313–388 g). The rats were ≈4 months old at the start of the experiments, and were housed in pairs in the colony room that was illuminated between the hours of 08.00 and 20.00, with food and water available *ad libitum* in the home cage throughout the experiment. Following a minimum of 2 weeks of postoperative recovery, rats received behavioral training that began at ≈09.30 on each day. All experimental procedures and animal husbandry conformed to the “principles of laboratory animal care” (Guide for the Care and Use of Laboratory Animals, NIH publication No. 85-23, revised 1985) and the UK Animals (Scientific Procedures) Act (1986), and received local ethics committee approval at Cardiff University.

### 2.2 Surgery and histology

The surgical procedure and the coordinates of injection sites were closely modeled on those described in Marshall, McGregor, Good, and Honey (2004). To summarize, rats were first anaesthetized with Isoflurane and then placed in a stereotaxic frame (David Kopf Instruments, Tujunga, CA). After scalp incision, the bone overlying the area of neocortex directly above the hippocampus was removed, and a 2-μl Hamilton syringe mounted on the stereotaxic frame was used to infuse ibotenic acid into the hippocampus. The ibotenic acid (supplied by Biosearch Technologies, San Rafael, CA) was dissolved in phosphate-buffered saline [pH 7.4] providing a solution with a concentration of 63 mM; and injections of 0.05–0.10 μl were made at 30 sites with a KD Scientific electronic pump (Model 5000; Boston, MA) at a rate of 0.05 μl/min (see Table 2). After each injection, the needle was left in position for 2 min to allow diffusion of the ibotenic acid and to limit the spread of the drug into overlying cortical areas. Sham-operated rats received an identical treatment with the exception that dura was perforated with a 25-gauge Microlance3 needle (Becton Dickinson, Drogheda, Ireland) and no fluid was infused.

After behavioral testing, rats received a lethal overdose of sodium pentobarbitone (Euthatal), and were then transcardially perfused, first with 0.9% saline and then with 10.0% formal-saline. Their brains were then extracted and postfixed for 24 h, and transferred to phosphate-buffered (0.1 M) 25.0% sucrose solution for 24 h. Subsequently, each brain was frozen, sectioned coronally using a −20 °C cryostat, and the 40 μm sections were collected on gelatine-coated slides. These slides were left to dry at room temperature for 24 h, and then stained with cresyl violet. The sections were examined using a microscope, and histological borders of hippocampal lesions were verified with reference to the boundaries defined by Paxinos and Watson (2005).

### 2.3 Apparatus

The apparatus used was that described in Lin et al. (2013) and consisted of 8 operant chambers (Test chamber 80004-D001; Campden Instruments Ltd., Loughborough, England; 30.5 cm × 26 cm × 20 cm; width × depth × height) arranged in 4 × 2 array. Each chamber was housed within a sound-attenuating shell, had two aluminum side walls, a transparent Perspex back wall and ceiling. The front wall was also Perspex, and served as the door to the chamber. The chambers were lit by a 3-W light bulb, with a white plastic cover, positioned centrally and 13.5 cm above the floor. Two additional visual stimuli served as A and B: illumination of covered 3-W jewel lights that were located on the left- and right-hand sides of the left aluminum wall that contained the food well. These lights were mounted 13.5 cm above the floor and were positioned 9.2 cm to the left and right of a central wall light that was unused and mounted at the same height above the floor but immediately above the food well. The left and right lights were both constantly illuminated throughout their 30-s durations. Two 30-s auditory stimuli served as X and Y: a 2-kHz tone and a 2-Hz clicker. These stimuli, presented at an intensity of ≈75 dB, were produced by an internal audio generator and delivered through a speaker located centrally and at 14.5 cm above the floor on the left aluminum wall. A grid constructed from 19 stainless steel bars (diameter 0.47 cm, spacing from bar center to bar center, 1.07 cm) served as the floor of the chamber. A 0.5-s 0.64 mA footshock could be delivered through the grid floor.

During conditioning, the levels of activity during X and Y was similar and did not differ in either group. However, we also measured activity during the 40-s trace periods after X and Y and these scores represent the focus of our analysis of the effectiveness of conditioning in Experiments 1 and 2. In both experiments, test performance was assessed through the levels of activity during presentations of test compounds (Experiment 1: AX, BX, AY, BY) or the elements (Experiment 2: A and B); and during the 60-s periods that preceded each test stimulus. Activity was measured using an automated ambulatory monitor consisting of two horizontal strips, one attached to the front wall (i.e., the door) and the other attached to the back wall. These strips were positioned 3.0 cm above the grid floor, and each contained three infrared light sources and photobeam detectors that were located 3.0 cm from the left-hand wall, in the center of the chamber and 3.0 cm from the right-hand wall. Detection of the presence of the rat in the area covered by a photobeam followed by detection of the absence of the rat in this area, as the rat moved in the chamber, was recorded as a value of 1. The number of times this occurred for each of the three beams provided a single value for the total movement made by the rat in the chamber. It was assumed that lower levels of activity were indicative of greater fear during the final test. Previous experiments have established the utility of this measure in providing automated measures of standard behavioral effects based on fear conditioning (see Dumigan et al., 2015, Lin et al., 2013). A computer (Mark II Control Unit) controlled the apparatus, operated the program (using Behavioural Net Controller Control 1.0) and recorded ambulatory movement (all equipment was supplied by Campden Instruments Ltd., Loughborough, England).

### 2.4 Behavioral procedure

The procedure was modeled on that described in Lin et al. (2013). Rats in Experiments 1 and 2 received variants of sensory preconditioning procedure that each involved 3 stages: preexposure, conditioning and test (see Table 1). For rats in both Experiments 1 and 2, the preexposure stage consisted one session per day for 6 days (days 1–6). In each session, there were two types of 30-s simultaneous compound: AX (e.g., the left light presented with the tone) and BY (e.g., the right light presented with the clicker). For half of the rats in each group (Sham and HPC), the left light served as A and the right light served as B, and for the remainder the reverse was the case. In the subgroups created by the previous counterbalancing operation, for half of the rats the tone served as X and the clicker served as Y and for the remainder the reverse was the case. There were 10 presentations of each compound per session, that were presented in a pseudorandom order with the constraint that there were no more than two trials of the same type in succession. The intertrial interval (ITI) was 2.5 min.

In both experiments we used the same trace conditioning procedure, because this procedure results in the theoretically important difference between AX and BX during the test (see Lin et al., 2013). It has been argued that a trace conditioning procedure might be particularly conducive to observing mediated learning involving an evoked memory (Ward-Robinson & Hall, 1996). Moreover, it has been established that the trace interval used here has equivalent effects on rats with lesions of the hippocampus as it does on rats with sham lesions (see Lin & Honey, 2011). In both experiments, rats received 2 conditioning sessions (on days 7 and 8), one session per day. In each session, rats received 3 presentations of X (e.g., tone) followed by footshock after a 40-s trace interval and 3 presentations of Y that were not followed by footshock (i.e., X-trace-footshock, Y-no footshock). For half of the rats, the sequence was XYYXYX, and the rest received YXXYXY, with an ITI of 8 min. During the critical two test days, rats in Experiment 1 received tests with four configurations: AX, BX, AY and BY. For half of the rats, Test 1 (involving a comparison of AX versus BX) was on day 1 and Test 2 (involving AY versus BY) was on day 2; and for the remainder the order of Test 1 and Test 2 was reversed. For half of the rats on each day, the order of trials in Test 1 was AX, BX, BX and AX, and for the remainder it was BX, AX, AX and BX. Similarly, for half of the rats on each day, the order of trials was AY, BY, BY and AY, and for the remainder it was BY, AY, AY and BY. These tests allow retrieval-mediated learning involving AX to be assessed (e.g., by comparing AX and BX). In Experiment 2, rats received presentations of A and B during the tests in a counterbalanced order: half of the rats received A, B, B, A, B and A, and the remainder received B, A, A, B, A and B. The ITI during the tests in experiments 1 and 2 was 8 min and each test stimulus or compound was presented for 60 s.

## 3 Results

### 3.1 Histology

**Fig. 1:**
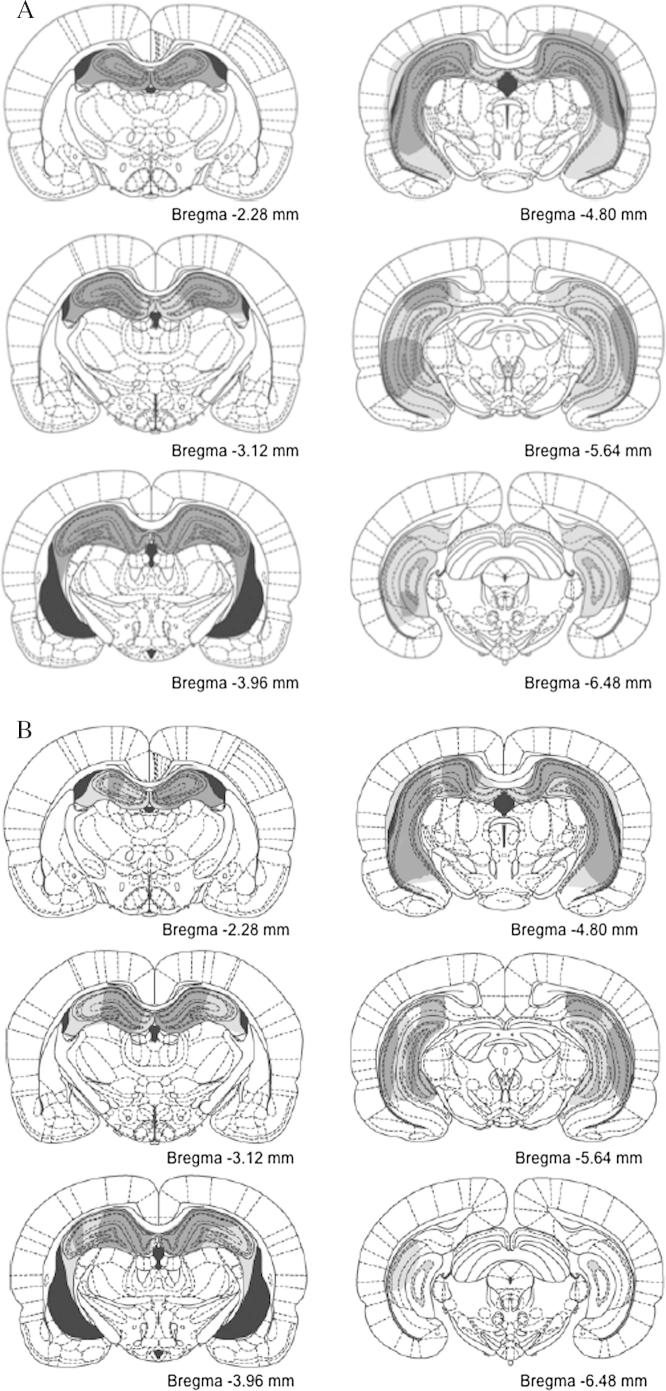
(A and B) The maximum (light gray) and minimum (dark gray) extent of the lesions in rats in group HPC in Experiment 1 (A) and Experiment 2 (B). The coronal sections are at specific distances (in mm) from Bregma (top left to bottom right: −2.28, −3.12, −3.96, −4.80, −5.64, −6.48; adapted from Paxinos & Watson, 2005).

Fig.1A and B depict a series of coronal sections through the rat brain (adapted from Paxinos & Watson, 2005), with the largest overall lesion (in light gray) and the smallest lesion (in dark gray) in Experiments 1 (A) and 2 (B). Inspection of the panels in both figures suggests that the lesions were similar in the two experiments. To estimate the extent of hippocampal damage all lesions were plotted onto six equally spaced, coronal sections (Bregma −2.28, −3.12, −3.96, −4.80, −5.64, −6.48; adapted from Paxinos & Watson, 2005). All 16 rats in Group HPC in Experiments 1 and 2 had acceptable extensive bilateral lesions. Assessments of total hippocampal tissue damage revealed variability in both experiments (Experiment 1: range 60.3–98.2%; Experiment 2: range 53.5–71.62%; see Broadbent, Squire, & Clark, 2004). The tissue loss in the dorsal (septal) hippocampus was particularly extensive (dentate gyrus, CA1-4), with all cases in Experiment 1 and seven out of eight cases in Experiment 2 having >75% loss. [For this analysis the border between dorsal and ventral hippocampus was arbitrarily placed at −5.5 below bregma (Paxinos & Watson, 2005).] In both experiments, the damage to the ventral hippocampus was more limited and variable. The lesions typically involved most of the CA1 and CA3 subfields in the ventral hippocampus, though remnants of the ventral dentate gyrus were present. Damage was limited to the hippocampus in most cases, with two rats in Experiment 1 having minor cortical damage to the primary motor cortex.

### 3.2 Behavior

#### 3.2.1 Conditioning

The upper panel of Table 3 shows the difference between the levels of activity on the first and the final trials during the trace periods after X and Y for groups Sham and HPC in Experiment 1. Inspection of this panel suggested that the reduction in activity was greater during the trace of X than Y in groups Sham and HPC. ANOVA with group and stimulus as factors confirmed that there was a main effect of stimulus, *F*(1, 14) = 5.67, *p *< 0.05, but there was no effect of group and no interaction between these two factors, *F*s < 1.

The lower panel of Table 3 shows the corresponding scores from Experiment 2. Inspection of this panel suggested that the reduction in activity was greater during the trace of X than Y; and that this difference was similar in groups Sham and HPC. ANOVA with group and stimulus as factors confirmed that there was a main effect of stimulus, *F*(1, 14) = 6.805, *p *< 0.05, but there was no effect of group and no interaction between these two factors, largest *F*(1, 14) = 2.31, *p *> 0.15.

#### 3.2.2 Test

**Fig. 2:**
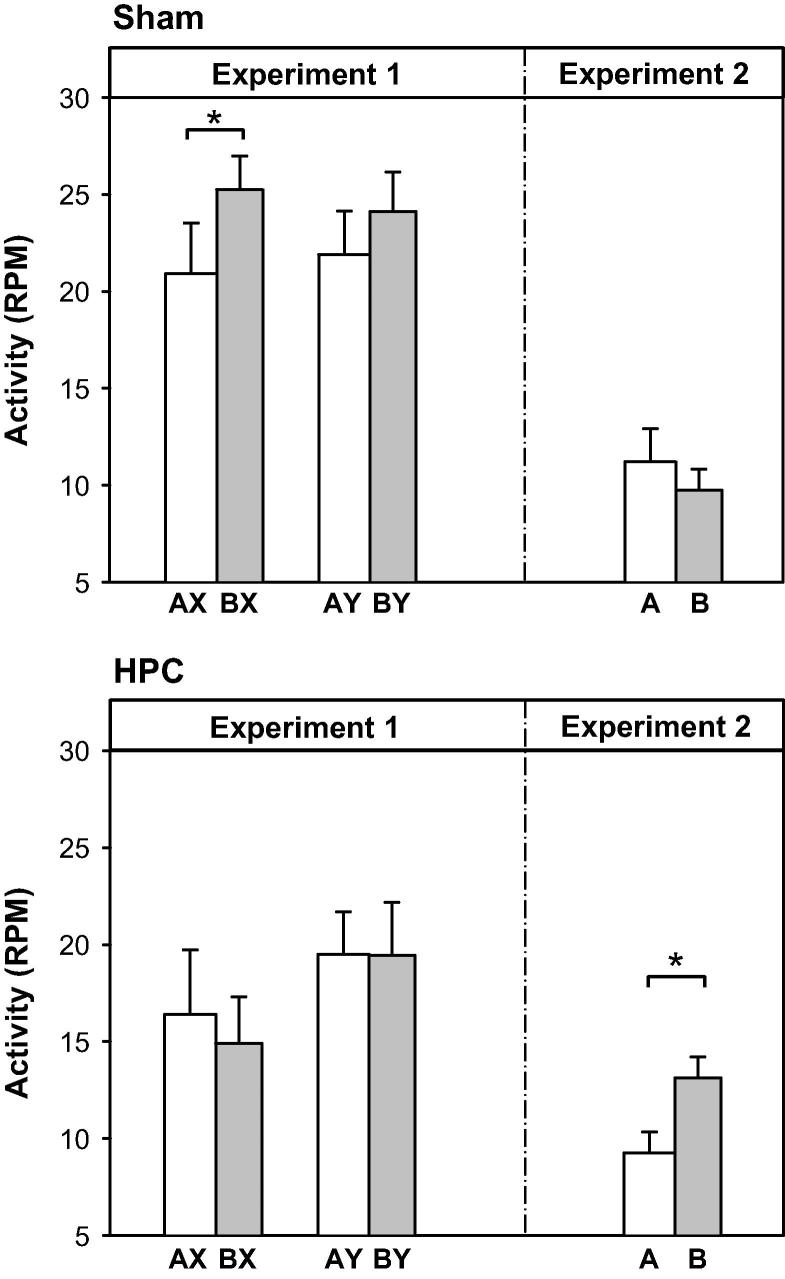
Mean activity levels (in responses per minute, RPM; +SEM) during the test with AX and BX (and AY and BY; Experiment 1; left panels), and A and B (Experiment 2; right panels). Rats in groups Sham (upper panels) and HPC (lower panels) had received exposure of AX and BY prior to trials on which X was followed by shock after a trace interval of 40 s and Y was not. Asterisks indicate *p* < 0.05.

The mean activity levels during the tests in Experiments 1 and 2 are shown in the left and right panels of Fig. 2, respectively. The results for group Sham are shown in the upper panel while those for group HPC are presented in the lower panel. We consider first the test trials given to rats in Experiment 1. These patterns (AX, BX, AY and BY) clearly differ in their similarity to the configuration (AX) that should have been stored during preexposure and activated on the trace conditioning trials with X: AX most closely matches this configural trace, whereas BX is less similar to it. However, it should be noted that the comparison involving compounds that differ from AX in terms of the absence of either A or X (i.e., comparing BX with AY) is complicated by the fact that A and B were visual stimuli and X and Y were auditory (and the similarity of A to B and X to Y not known). In any case, rats in group Sham showed less activity (i.e., more fear) during AX than BX and, to lesser degree, less activity during AY than BY; and these rats showed little difference in activity between compounds containing X and Y. This pattern of results was not evident in rats in group HPC, who showed no difference between AX and BX (or AY and BY). ANOVA with group (Sham and HPC), presence of directly conditioned stimulus (X or Y) and nature of configuration (containing A, AX and AY, or containing B, BX and BY), revealed no effect of group, *F*(1, 14) = 3.53, *p *> 0.08, conditioned stimulus, *F*(1, 14) = 1.93, *p *> 0.18, or configuration, *F*(1, 14) = 1.78, *p *> 0.20; but there was an interaction between group and configuration, *F*(1, 14) = 4.72, *p *< 0.05, and no other interactions, largest *F*(1, 14) = 2.09, *p *> 0.17. Analysis of simple main effects confirmed an effect of configuration in group Sham, *F*(1, 14) = 6.15, *p *< 0.05, but not group HPC, *F *< 1.

Supplementary analyses of the results from the two types of test (involving AX versus BX, and AY versus BY) were conducted. The analysis of the test involving AX and BX showed that there was an effect of group, *F*(1, 14) = 4.72, *p *< 0.05, no effect of configuration, *F*(1, 14) = 1.32, *p *> 0.27, and an interaction between these factors, *F*(1, 14) = 5.64, *p *< 0.05. Analysis of simple main effects revealed a difference between AX and BX in group Sham, *F*(1, 14) = 6.21, *p *< 0.05, but not in group HPC, *F *< 1. A parallel analysis of the comparison between AY and BY revealed no effect of group or configuration and no interaction between the two, largest *F*(1, 14) = 1.39, *p *> 0.25. The mean rates of responding during 60-s periods that immediately preceded the test stimuli were somewhat higher in group Sham (*M* = 17.02 rpm, *SEM* = 1.24) than in group HPC (*M* = 12.88, *SEM* = 2.06), but this difference was not statistically significant, *F*(1, 14) = 2.96, *p* > .10.

The right-hand panels of Fig. 2 depict the levels of activity during A and B in the final test of Experiment 2. It was noted that there was very little activity recorded in the zone covered by the left beam and the analysis was therefore restricted to the center and right beams. ANOVA with stimulus (A and B) and group (Sham and HPC) as factors revealed there was no effect of stimulus or group, largest *F*(1, 14) = 1.01, *p *> 0.33, but the interaction between stimulus and group was significant, *F*(1, 14) = 4.91, *p *< 0.05. Analysis of simple main effects confirmed that the levels of activity during A was lower than during B in group HPC, *F*(1, 14) = 5.19, *p *< 0.05, but not in group Sham, *F *< 1.^1^ The mean rates of responding during 60-s periods that immediately preceded the test stimuli were similar in groups Sham (*M* = 14.19 rpm, *SEM* = 1.37) and HPC (*M* = 14.91, *SEM* = 1.84), and, as in Experiment 1, did not differ significantly, *F *< 1.

Experiments 1 and 2 were closely matched, aside from the nature of the test stimuli (compounds in Experiment 1 and elements in Experiment 2), and yet the levels of activity were generally lower in Experiment 2 than Experiment 1. This observation is most likely to reflect a difference in unconditioned suppression of activity in the two types of test. For example, the test in Experiment 2 was the first time that the rats experienced A and B alone and this might have resulted in unconditioned suppression of activity. Alternatively, the presentation of X and Y in Experiment 1 might generate unconditioned activity either directly or by interacting with the visual stimuli (A and B).

## 4 General discussion

We employed novel sensory preconditioning procedures to assess the role of the hippocampus in pattern completion (e.g., Leutgeb et al., 2007, Rolls, 2013). After exposure to two audio-visual patterns (AX and BY) rats received trace conditioning with X (but not Y) and then critical tests with AX and BX (Experiment 1) and A and B (Experiment 2). In Experiment 1, rats in group Sham showed more evidence of fear during AX than BX, and this difference was less apparent during AY and BY. The observation that AX elicited less activity than BX might reflect the fact that AX was a familiar compound and BX was a novel compound (e.g., Honey and Good, 2000, Honey et al., 1998, Honey et al., 1998). However, if this was the sole determinant of test performance then the familiar compound BY should have also provoked less activity than the novel compound AY, and this was not the case. Instead the pattern of results observed in group Sham in Experiment 1 is consistent with the suggestion that during conditioning with X the configural memory of AX was linked to footshock; or with the more general possibility that the memory activated during conditioning more closely matches AX than BX (cf. Lin et al., 2013). Further support for this interpretation comes from the observation that the difference at test between AX and BX is also evident even when both X and Y have been paired with shock (Lin et al., 2016). There was no evidence of a difference in fear to AX and BX in group HPC. This deficit could reflect a failure to encode the compounds during preexposure (or test) or a failure of conditioning. The results of Experiment 2 undermine these forms of explanation. In Experiment 2, rats in group HPC showed more fear to A than to B, which indicates both that they had linked the elements of each compound and that the conditioning procedure, used in both Experiments 1 and 2, was effective. The observation that in group Sham there was no difference in fear between A and B means that we have a clear double dissociation that prompts the obvious question: Why does the nature of the test involving A and B (in compound with X or alone) affect whether the they will provoke different levels of fear in groups Sham and HPC?

We have argued that there is a straightforward answer to this question in the context of the behavior of rats in group Sham. Exposure to AX will allow a representation of this compound to be formed that becomes linked to shock during conditioning; and test stimuli will elicit fear to the extent that they match this AX representation. Clearly, the presentation of AX is more similar to this representation than is either BX or critically A. What then of the pattern of results observed in group HPC? The assumption that the processes just outlined are undermined in rats with hippocampal lesions should only lead one to expect that there should be no difference between AX and BX, not that this effect should be accompanied by a difference between A and B that is not evident in control rats. An additional assumption is clearly required. One possibility is to appeal to the idea that learning about AX during conditioning ordinarily disrupts or overshadows learning about X itself (cf. Ward-Robinson & Hall, 1996). This will mean that that any A-X-shock associative chain will be more likely to contribute to the fear that A elicits in group HPC, for which mediated AX learning is disrupted, than in group Sham: because the X-shock association will be stronger in group HPC than in group Sham. Indeed there was some evidence that the levels of fear to the compounds containing X were greater in group HPC than in group Sham.

It should be noted that the theoretical analysis just outlined does not require that the hippocampus is involved in configural processes in general, even when the potential for compensatory propositional strategies that humans can use are unlikely to be an issue (see Ryan, Moses, Barense, & Rosenbaum, 2013; cf. Shanks & Darby, 1998). Thus, it is well established that rats with damage to the hippocampus are not routinely impaired in acquiring reinforced discriminations that require configural processes (e.g., Coutureau et al., 2002, Saksida et al., 2007). Instead our analysis assumes that the hippocampus underpins mediated learning involving configural representations established during simple exposure. There is independent evidence that is consistent with this specific claim from a recent series of studies (Iordanova et al., 2009, Iordanova et al., 2011, Iordanova et al., 2011). Thus, rats first received exposure to four patterns involving the components of episodic memory (what happened where and when): a tone was presented in a spotted context in the morning and a checked context in the afternoon, and a clicker was presented in the checked context in the morning and the spotted context in the afternoon. After pairing the tone with footshock at midday in a third context, rats showed more fear to the spatio-temporal configurations in which the tone had been previously presented (i.e., the spotted context in the morning and the checked context in the afternoon) than in the configurations in which the clicker had been presented. This effect was abolished in rats with lesions of the hippocampus (Iordanova et al., 2009, Iordanova et al., 2011) and by infusing AP5 (an NMDA receptor antagonist) into the hippocampus during fear conditioning (Iordanova, Burnett, et al., 2011). In this context, it is interesting to note that in a recent fMRI study using a SPC procedure with visual stimuli in humans, Wimmer and Shohamy (2012) presented evidence suggesting that their SPC effect (at test) was correlated with hippocampal activity during the equivalent of the conditioning stage of the study, but not the exposure or test stage. The results of Experiments 1 and 2, together with those of Wimmer and Shohamy (2012), suggest that the involvement of the hippocampus in retrieval-mediated learning is not restricted to configurations involving episodic content, and indicate that configural and elemental processes of pattern completion operate in concert and interact (cf. Honey et al., 2014).

Our new results provide general support for the view that the hippocampus underpins aspects of pattern completion (e.g., Leutgeb et al., 2007, Rolls, 2013). They specifically indicate that the hippocampus is involved in configural processes, and in doing so have highlighted the need to appeal to interacting configural and elemental processes of pattern completion (e.g., Honey et al., 2014, Sutherland and Rudy, 1989).

## Figures and Tables

**Table 1:** Design of Experiments 1 and 2.

*Note*: Both Experiments 1 and 2 involved three stages: preexposure, conditioning and test. Rats in groups Sham and HPC received preexposure to two audio-visual compounds (AX and BY) before trace conditioning trials with X and nonreinforced presentations of Y. Rats in Experiment 1 then received tests with the compounds AX and BX (and AY and BY); while those in Experiment 2 received tests with A and B alone.

**Table 2:** Stereotaxic coordinates and volume of ibotenic acid for lesions of the hippocampus.

	AP	ML	DV	Volume (μl)
From bregma:	−5.5	±4.2	−7.6	0.10
−3.9	0.10
±5.5	−6.8	0.10
−5.8	0.10
−5.0	0.10
−4.7	±4.0	−7.5	0.10
−3.5	0.05
±4.5	−8.0	0.10
−3.9	±2.2	−3.7	0.10
−3.0	0.10
±3.5	−2.7	0.10
−3.1	±1.4	−4.0	0.10
−3.0	0.10
±3.0	−2.7	0.10
−2.4	±1.0	−3.8	0.05

*Note*: AP, ML and DV indicate the coordinates in relation to bregma from anterior to posterior (AP), from medial to lateral (ML) and from dorsal to ventral (DV).

**Table 3:** Experiments 1 and 2: Mean difference in activity scores in rpm (+SEM) between the first and final conditioning trials during the trace periods that followed X and Y.

	X	Y
Experiment 1:		
Sham	−17.91 (4.01)	−8.25 (2.71)
HPC	−15.66 (3.82)	−8.16 (3.75)

Experiment 2:		
Sham	−12.75 (2.64)	−1.93 (1.58)
HPC	−9.62 (3.77)	−6.77 (3.07)

## Footnotes

1 Given the lack of evidence of SPC in group Sham, we ran a replication of Experiment 2 with identical procedures. The same pattern of test results was observed in this replication: Sham A = 19.57 rpm (1.97) and B = 15.44 rpm (1.38); and HPC A = 13.89 rpm (0.95) and B = 17.59 (1.35). ANOVA with group and stimulus as factors revealed there was no effect of group or stimulus, largest *F*(1, 23) = 2.63, *p *> 0.11, but a significant interaction between group and stimulus, *F*(1, 23) = 11.50, *p *< 0.01. Analysis of simple main effects confirmed that the levels of activity during A were lower than during B in group HPC, *F*(1, 23) = 7.33, *p *< 0.05, but not in group Sham, *F*(1, 23) = 4.18, *p *> 0.05.
